# Predictive Power of EEG in Infants with Tuberous Sclerosis

**DOI:** 10.15844/pedneurbriefs-30-2-1

**Published:** 2016-02

**Authors:** Molly Tracy

**Affiliations:** 1 Departments of Neurology and Pediatrics and Neurology, Brown University, Providence, RI

**Keywords:** Epileptic Spasms, EEG, Tuberous Sclerosis

## Abstract

Investigators from University of California- Los Angeles and collaborators from across the country report on the use of prospective EEGs in infants with tuberous sclerosis complex (TSC) to predict evolution to seizures.

Investigators from University of California- Los Angeles and collaborators from across the country report on the use of prospective EEGs in infants with tuberous sclerosis complex (TSC) to predict evolution to seizures. They prospectively enrolled infants with TSC in an observational study which included regularly scheduled exams and research one-hour video EEGs. These infants were diagnosed on the basis of clinical features (i.e. cardiac rhabdomyomas, intracranial MRI findings, skin findings) or genetic diagnosis. The infants were younger than 7 months at enrollment, were seizure free, and had not been treated with TS specific medications (vigabatrin or inhibitors of the mammalian target of rapamycin [mTOR].) If the child developed seizures, EEG was performed to confirm the diagnosis and an appropriate anti-epileptic drug was initiated. This was an interim publication of findings, with 28/40 enrolled patients older than a year at the time of analysis. Of those, 19 (67.8%) had developed seizures, with epileptic spasms being the most frequent seizure type (52.6%.) All children with epileptiform discharges on EEG went on to develop epilepsy (100% positive predictive value). [1]

COMMENTARY. This study suggests that serial EEGs may be used as a biomarker for subsequent epilepsy in infants with early diagnosis of TSC [1].

TSC is an autosomal dominant hamartomatous condition affecting various organ systems, but most consistently presenting with CNS symptoms. These may include epilepsy (85%), intellectual disability or other neuropsychiatric condition. The genes implicated code for hamartin and tuberin (TSC1 and 2), which control cell proliferation through mTOR pathways in cells. CNS lesions may include subependymal nodules, cortical tubers, and subependymal giant cell astrocytomas. Cardiac rhabdomyomas may be detected on second trimester ultrasounds and lead to prenatal diagnosis. Renal angiomyolipomas and renal cysts are also prevalent. Two- thirds of infants with TSC develop seizures before a year of age, and earlier seizures lead to more significant neuropsychiatric sequelae [2].

Increased mTOR activity is believed to increase epileptogenicity in multiple ways, including leading to dysplastic neurons, abnormal astrocytes, loss of normal cortical structure, abnormal dendritic arborization, decreased GABAergic inhibition and increasing glutamatergic excitation. Because of specific effects of GABA systems and some inhibition of mTOR signaling pathways, vigabatrin is first line treatment for infantile spasms and focal seizures in TSC patients before a year of age. Early diagnosis of seizures and initiation of vigabatrin is particularly important for intellectual development and achieving seizure control [3]. The referenced article from Wu et al suggests that interictal epileptiform discharges may be detectable prior to seizure onset in many children with TSC and their presence reliably predicts the later development of seizures [1]. In the future mTOR inhibitor treatment may be initiated when EEGs indicate epileptogenicity and prior to seizure onset to improve neurodevelopmental outcomes and long-term prognosis of epilepsy in patients with TSC, however studies are still in progress [4].
